# Fatores de Confundimento na Análise da Relação entre a Calcificação do Arco Aórtico com o Padrão de Pressão Arterial não Dipper

**DOI:** 10.36660/abc.20200750

**Published:** 2021-01-27

**Authors:** Pedro Pereira Tenório, Carlos Alberto de Lima Botelho, Romero Henrique de Almeida Barbosa, Johnnatas Mikael Lopes

**Affiliations:** 1 Universidade Federal do Vale do São Francisco Paulo AfonsoBA Brasil Universidade Federal do Vale do São Francisco - Colegiado de Medicina, Paulo Afonso, BA - Brasil

**Keywords:** Aorta Torácica, Calcificação, Pressão Arterial, Razão de Prevalências, Tórax/radiografia

**Caro Editor,**

Lemos com grande interesse o artigo “A Calcificação no Arco Aórtico na Radiografia de Tórax de Rotina está Forte e Independentemente Associada ao Padrão Não-Dipper de Pressão Arterial”. Nesse estudo, objetivou-se avaliar a possível relação entre a calcificação do arco aórtico, na radiografia de tórax, com o padrão de pressão arterial não dipper. Foram analisados 406 pacientes divididos em dois grupos: dipper e não dipper. Cerca de 261 (64%) pacientes apresentavam o padrão de pressão arterial não dipper contra 145 (36%) dipper. Verificou-se que o grupo não dipper apresentou maior prevalência de calcificação do arco aórtico (70% vs. 33%, p<0.0001).

Na análise multivariada do estudo, o desfecho de interesse é se o participante pertence ao grupo não dipper a partir de uma variável dicotômica. Por se tratar de um desenho transversal e não de caso-controle, pois as variáveis independentes não são retrospectivas e não há pareamento dos grupos, a estratégia mais indicada de análise seria regressão de Poisson ou Cox. Diferentemente da regressão logística que tem como medida de efeito o *Odds Ratio* (OR), a regressão de Poisson e Cox estimam a Razão de Prevalência (RP), cuja aplicação é mais apropriada ao desenho. OR e RP serão apenas semelhantes quando os desfechos são pouco frequentes (<10%).^1^

O uso da OR nesse estudo traz possíveis vieses devido a alta prevalência do desfecho (PADV), fazendo com que a estimativa pontual fique superdimensionada e o seu intervalo de confiança mais dilatado. Essa condição de análise traz dúvidas para variáveis como a idade, índice de massa corporal, índice de massa ventricular esquerda, triglicerídeos e a taxa de filtração glomerular, que tem intervalos de confiança muito limítrofes (OR~1). É muito provável que a calcificação esteja associada à PADV, mas não sozinha e/ou em menor intensidade.^2^

Cerca de 59 (22,6%) dos pacientes do grupo não dipper e 25 (17,2%) do grupo dipper eram diabéticos. Os pesquisadores não indicaram que tipo de diabetes os pacientes possuíam no grupo não dipper e se tais pacientes eram ou não resistentes à insulina (RI). Sabe-se que a (RI) conduz a níveis plasmáticos elevados de insulina e que esta atua a nível dos receptores hipotalâmicos do sistema nervoso central (SNC), levando a um aumento do fluxo simpaticomimético.^3 , 4^ Dessa forma, há predomínio da atividade simpática. Vários estudos demonstraram que a ativação simpática é diretamente proporcional à gravidade da hipertensão arterial. Assim, nas formas mais graves de hipertensão arterial, o fluxo simpaticomimético é mais proeminente.^3^ Os autores poderiam ter avaliado a real influência da RI nos diabéticos do grupo não dipper com o objetivo de identificar a real atuação da diabetes, evitando um fator de confundimento, pois não é possível afirmar se tais pacientes apresentaram o padrão de pressão não dipper devido a RI ou a outros fatores.

Outra consideração importante é que o estudo não faz menção a algumas limitações da Monitorização Ambulatorial da Pressão Arterial (MAPA), considerando-se que não foi feita a avaliação da qualidade do sono dos pacientes. É sabido que uma baixa qualidade do sono associado ao nível de desconforto relacionado ao método pode interferir significativamente no descenso noturno da pressão arterial. Além disso, não foram excluídos da pesquisa pacientes portadores de arritmia como fibrilação atrial, *flutter* atrial e extra-sístoles ventriculares frequentes,^5^ podendo, assim, também serem importantes fatores de confusão na correlação entre a calcificação no arco aórtico com o padrão não dipper avaliado pela MAPA.
