# Supplementary material for: Fatores de Confundimento na Análise da Relação entre a Calcificação do Arco Aórtico com o Padrão de Pressão Arterial não Dipper
Source: Arq Bras Cardiol. 2021 Jan 27;116(1):158–9. [Article in Portuguese] doi: 10.36660/abc.20200750 (PMC8159514; doi:10.36660/abc.20200750)
Supplement: Translation

# Translation: Confounding Factors in the Analysis of the Relationship between Aortic Arch Calcification with a Non-Dipper Blood Pressure Pattern

**Authors:** Pedro Pereira Tenório, Carlos Alberto de Lima Botelho, Romero Henrique de Almeida Barbosa, Johnnatas Mikael Lopes

**Affiliations:** 1 Brazil Universidade Federal do Vale do São Francisco - Colegiado de Medicina, Paulo Afonso, BA - Brazil

**Keywords:** Aorta, Thoracic, Calcification, Blood Pressure, Prevalence Ratio, Thorax/radiography

**Dear Editor,**

We read the following article with great interest: “Aortic Arch Calcification on Routine Chest Radiography is Strongly and Independently Associated with Non-Dipper Blood Pressure Pattern”. In this study, the aim was to evaluate a possible relationship between aortic arch calcification on chest radiographs and the non-dipper blood pressure pattern. Altogether, 406 patients were analyzed and divided into two groups: dipper and non-dipper. Approximately 261 (64%) patients presented the pattern of non-dipper blood pressure, against 145 (36%) with dipper blood pressure. It was found that the non-dipper group presented a higher prevalence of aortic arch calcification (70% vs. 33%, p<0.0001).

In the multivariate analysis of the study, the outcome of interest is whether the participant belongs to the non-dipper group from a dichotomous variable. As this is a cross-sectional design and not a case-control design, since the independent variables are not retrospective and there is no pairing of the groups, the most indicated analysis strategy would be Poisson or Cox regression. Unlike logistic regression that has Odds Ratio (OR) as a measure of effect, Poisson and Cox regression estimate the Prevalence Ratio (PR), whose application is more appropriate to the design. OR and PR will only be similar when the outcomes are infrequent (<10%).^1^

The use of OR in this study brings potential bias due to the high prevalence of the outcome (PADV), making the point estimate over dimensional and its confidence interval more dilated. This condition of analysis brings doubts for variables such as age, body mass index, left ventricular mass index, triglycerides and the glomerular filtration rate which has very borderline confidence intervals (OR~1). It is very likely that calcification is associated with PADV, but not alone and/or to a lesser extent.^2^

Nearly 59 (22.6%) of the patients in the non-dipper group and 25 (17.2%) in the dipper group were diabetics. The researchers did not indicate what kind of diabetes the patients had in the non-dipper group and whether or not these patients were insulin resistant (IR). It is known that IR leads to high plasma levels of insulin and that it acts at the level of hypothalamic receptors of the central nervous system (CNS), leading to an increase in sympathomimetic flow.^3 , 4^This way, there is a predominance of sympathetic activity. Several studies have shown that sympathetic activation is directly proportional to the severity of hypertension. Thus, in the most severe forms of hypertension, the sympathomimetic flow is more prominent.^3^The authors could have evaluated the real influence of IR on diabetics of the non-dipper group in order to identify the real action of diabetes, avoiding a confounding factor, because it is not possible to state whether such patients had the pattern of non-dipper pressure due to IR or other factors.

Another important consideration is that the study does not mention some limitations of Ambulatory Blood Pressure Monitoring (ABPM), considering that the patients’ sleep quality was not evaluated. It is known that low quality of sleep associated with the level of discomfort related to the method can significantly interfere with nightly blood pressure drop. Besides, patients with arrhythmia were not excluded from the study, such as atrial fibrillation, atrial flutter and frequent ventricular extrasystoles.^5^Thus, the correlation between calcification in the aortic arch and the non-dipper pattern assessed by ABPM could also be important confounding factors.

# Translation: Reply

**Authors:** Pedro Pereira Tenório, Carlos Alberto de Lima Botelho, Romero Henrique de Almeida Barbosa, Johnnatas Mikael Lopes

**Affiliations:** 1 Brazil Universidade Federal do Vale do São Francisco - Colegiado de Medicina, Paulo Afonso, BA - Brazil

## Dear Editor,

Thank you for giving us the opportunity to respond to the comments and valid points raised by the authors. We would also like to thank the authors for their interest in and constructive comments on our paper.^1^

In this cross-sectional study, we investigated the potential relationship between aortic arch calcification (AAC) and non-dipper blood pressure (NDBP) pattern. We agree that this relationship could be evaluated with Cox regression analysis. However, we think that logistic regression analysis is also a suitable statistical analysis method for the study.^2 , 3^When Cox regression analysis was used instead of multiple regression analysis, and age, gender, hypertension, glomerular filtration rate, serum triglyceride level, left ventricular mass index, body mass index and AAC were taken as confounders, presence of AAC on chest radiography was again the only independent predictor of NDBP pattern (p≤0.001, HR=1.633 CI=1.215–2.194). These results were also confirmed with linear regression analysis. In linear regression analysis, the presence of AAC on chest radiography was associated with a lower systolic blood pressure drop at night.

Diabetes mellitus (DM) is known to be associated with NDBP pattern and insulin resistance is likely one of the most important etiopathogenetic pathways underlying this association.^4 , 5^In our study, DM was defined as being treated with insulin or oral hypoglycemic agents. Although there was no relationship between DM and NDBP pattern (p=0.201), we found a significant relationship between DM and AAC in this study (p=0.006). Since our main focus in this study was to investigate the potential relationship between AAC and NDBP pattern, we did not prioritize studying insulin resistance.

We agree that sleep quality may significantly affect nighttime blood pressure.^6^Night-shift workers were excluded from the study; however, we did not use any scale to quantify sleep quality in the study participants. Although we did not receive any negative feedback about sleep quality from any of the participants, we agree that the lack of evaluation of sleep quality is a limitation of the study.

Although all patients were in sinus rhythm at the time of enrollment, we cannot exclude the possibility of short atrial fibrillation/flutter episodes. Long-term rhythm monitorization is needed to detect paroxysmal arrhythmic episodes and to quantify the frequency of ventricular extrasystoles, which were beyond the scope of this study.

Once again, we would like to thank the authors for their thoughtful comments.
