# Supplementary material for: Fatores de Confundimento na Análise da Relação entre a Calcificação do Arco Aórtico com o Padrão de Pressão Arterial não Dipper
Source: Arq Bras Cardiol. 2021 Jan 27;116(1):158–9. [Article in Portuguese] doi: 10.36660/abc.20200750 (PMC8159514; doi:10.36660/abc.20200750)
Supplement: Related texts

# Reply: Carta-resposta

**Authors:** Pedro Pereira Tenório, Carlos Alberto de Lima Botelho, Romero Henrique de Almeida Barbosa, Johnnatas Mikael Lopes

**Affiliations:** 1 Universidade Federal do Vale do São Francisco Paulo AfonsoBA Brasil Universidade Federal do Vale do São Francisco - Colegiado de Medicina, Paulo Afonso, BA - Brasil

## Prezado Editor,

Agradecemos pela oportunidade de responder aos comentários e pontos válidos levantados pelos autores. Gostaríamos de agradecer aos autores pelo interesse em nosso estudo e pelos seus comentários construtivos.^1^

Nesse estudo transversal, investigamos a possível relação entre a calcificação do arco aórtico (CAA) e o padrão de pressão arterial não dipper (PAND). Concordamos que essa relação pode ser avaliada com a análise de regressão de Cox. No entanto, pensamos que a análise de regressão logística também é um método de análise estatística adequado para o estudo.^2 , 3^Quando a análise de regressão de Cox foi usada em vez da análise de regressão múltipla, e a idade, sexo, hipertensão, taxa de filtração glomerular, nível de triglicerídeos séricos, índice de massa ventricular esquerda, índice de massa corporal e CAA foram tomados como fatores de confusão, a presença de CAA na radiografia de tórax foi novamente o único preditor independente do padrão de PAND (p≤0,001, HR=1,633 CI=1,215–2,194). Esses resultados também foram confirmados com a análise de regressão linear. Na análise de regressão linear, a presença de CAA na radiografia de tórax esteve associada a menor queda da pressão arterial sistólica à noite.

Sabe-se que a diabetes mellitus (DM) está associada ao padrão PAND e a resistência à insulina é provavelmente uma das vias etiopatogenéticas mais importantes subjacentes a essa associação.^4 , 5^Em nosso estudo, a DM foi definida como sendo tratada com insulina ou hipoglicemiantes orais. Embora não tenha havido relação entre DM e o padrão PAND (p=0,201), encontramos uma relação significativa entre DM e CAA nesse estudo (p=0,006). Visto que nosso foco principal nesse estudo foi investigar a possível relação entre CAA e o padrão PAND, não priorizamos estudar a resistência à insulina.

Concordamos que a qualidade do sono pode afetar significativamente a pressão arterial noturna.^6^Trabalhadores noturnos foram excluídos do estudo; entretanto, não usamos nenhuma escala para quantificar a qualidade do sono nos participantes do estudo. Embora não tenhamos recebido nenhum feedback negativo sobre a qualidade do sono de nenhum dos participantes, concordamos que a falta de avaliação da qualidade do sono é uma limitação do estudo.

Embora todos os pacientes estivessem em ritmo sinusal no momento da inscrição, não podemos excluir a possibilidade de episódios curtos de fibrilação atrial/ *flutter* . É necessária a monitorização do ritmo a longo prazo para detectar episódios arrítmicos paroxísticos e quantificar a frequência de extrassístoles ventriculares, que estavam além do escopo desse estudo.

Mais uma vez, gostaríamos de agradecer aos autores pelo interesse em nosso estudo.

## References

[1] . Adar a, onalan o, cakan f, akbay e, karakaya e . Aortic arch calcification on routine chest radiography is strongly and independently associated with non-dipper blood pressure pattern. Arq bras cardiol. 2020;114(1):109-17.10.5935/abc.20190229PMC702529831664318

[2] . Yu cx, zhang xz, zhang k, tang z. A cross-sectional study for estimation of associations between education level and osteoporosis in a chinese men sample. Bmc musculoskelet disord. 2015 dec 09;16:382.10.1186/s12891-015-0839-0PMC467375226646811

[3] . Muniz dd, siqueira ks, cornell ct, fernandes-silva mm, muniz pt, silvestre om. Ideal cardiovascular health and job strain: a cross-sectional study from the amazon basin. Arq bras cardiol. 2019;112(3):260-8.10.5935/abc.20190005PMC642403930916204

[4] . Bauduceau b, mayaudon h, dupuy o, palou m, czerniak e, bredin c, et al. [the impact of dipper and non-dipper characteristics in the fluctuation of arterial blood pressure. A study of a population of 484 diabetic patients]. Arch mal coeur vaiss. 2000;93(8):969-73.10989739

[5] . Anan f, takahashi n, ooie t, yufu k, saikawa t, oshimatsu h. Role of insulin resistance in nondipper essential hypertensive patients. Hypertens res. 2003;26(9):669-76.10.1291/hypres.26.66914620920

[6] . Erden i, erden ec, ozhan h, basar c, aydin m, dumlu t, et al. Poor-quality sleep score is an independent predictor of nondipping hypertension. Blood press monit. 2010;15(4):184-7.10.1097/MBP.0b013e32833a23a020639701
